# CAR-T therapy pulmonary adverse event profile: a pharmacovigilance study based on FAERS database (2017–2023)

**DOI:** 10.3389/fphar.2024.1434231

**Published:** 2024-08-21

**Authors:** Jing Shi, Xinya Liu, Yun Jiang, Mengjiao Gao, Jian Yu, Yuanming Zhang, Li Wu

**Affiliations:** ^1^ Xinjiang Medical University, Urumqi, China; ^2^ Department of Oncology Cardiology, Xinjiang Medical University Cancer Hospital, Urumqi, China; ^3^ The Fifth Affiliated Hospital of Xinjiang Medical University, Urumqi, China

**Keywords:** chimeric antigen receptor T cells, CAR T cell therapy, FAERS database, real-world study, pharmacovigilance analysis, pulmonary adverse events

## Abstract

**Background:**

Chimeric antigen receptor T-cell (CAR-T) therapy, a rapidly emerging treatment for cancer that has gained momentum since its approval by the FDA in 2017, involves the genetic engineering of patients’ T cells to target tumors. Although significant therapeutic benefits have been observed, life-threatening adverse pulmonary events have been reported.

**Methods:**

Using SAS 9.4 with MedDRA 26.1, we retrospectively analyzed data from the Food and Drug Administration’s Adverse Event Reporting System (FAERS) database, covering the period from 2017 to 2023. The analysis included the Reporting Odds Ratio Proportional Reporting Ratio Information Component and Empirical Bayes Geometric Mean to assess the association between CAR-T cell therapy and adverse pulmonary events (PAEs).

**Results:**

The FAERS database recorded 9,400 adverse events (AEs) pertaining to CAR-T therapies, of which 940 (10%) were PAEs. Among these CAR-T cell-related AEs, hypoxia was the most frequently reported (344 cases), followed by respiratory failure (127 cases). Notably, different CAR-T cell treatments demonstrated varying degrees of association with PAEs. Specifically, Tisa-cel was associated with severe events including respiratory failure and hypoxia, whereas Axi-cel was strongly correlated with both hypoxia and tachypnea. Additionally, other CAR-T therapies, namely, Brexu-cel, Liso-cel, Ide-cel, and Cilta-cel, have also been linked to distinct PAEs. Notably, the majority of these PAEs occurred within the first 30 days post-treatment. The fatality rates varied among the different CAR-T therapies, with Tisa-cel exhibiting the highest fatality rate (43.6%), followed by Ide-cel (18.8%).

**Conclusion:**

This study comprehensively analyzed the PAEs reported in the FAERS database among recipients of CAR-T cell therapy, revealing conditions such as hypoxia, respiratory failure, pleural effusion, and atelectasis. These CAR-T cell therapy-associated events are clinically significant and merit the attention of clinicians and researchers.

## 1 Introduction

CAR-T cell therapy involves genetically modifying a patient’s T cells to recognize specific tumor antigens. These engineered cells are then cultured *ex vivo* and reinfused to launch a targeted attack against the patient’s cancer (Abramson et al.). Since the approval of the first chimeric antigen receptor T-cell (CAR-T) therapy, Tisagenlecleucel (tisa-cel), on 30 August 2017, the landscape of chimeric antigen receptor T-cell (CAR-T) products has evolved significantly. As of September 2023, the U.S. Food and Drug Administration (FDA) has granted approval to six CAR-T products. These products and their indications are listed in Table 1 (Munshi et al., 2021; Martin et al., 2023).

**TABLE 1:** FDA-Approved car-t cell therapies and indications.

Proper name	Abbreviated name	Indications
**Tisagenlecleucel**	**Tisa-cel**	• Pediatric and Young Adult B-cell Precursor Acute Lymphoblastic Leukemia (ALL) • Adult Large B-cell Lymphoma • Adult Follicular Lymphoma (FL)
**Axicabtagene-ciloleucel**	**Axi-cel**	• Adult Patients with Large B-cell Lymphoma • Relapsed or Refractory Large B-cell Lymphoma in Adults • Relapsed or Refractory Follicular Lymphoma (FL) in Adults
**Brexucabtagene autoleuce**	**Brexu-cel**	• Adult Patients with Relapsed or Refractory Mantle Cell Lymphoma (MCL) • Adult Patients with Relapsed or Refractory B-cell Precursor Acute Lymphoblastic Leukemia (ALL)
**Lisocabtagene maraleucel**	**Liso-cel**	• Adult Patients with Large B-cell Lymphoma • Chronic Lymphocytic Leukemia or Small Lymphocytic Lymphoma in Adults • Relapsed or Refractory Follicular Lymphoma in Adults • Relapsed or Refractory Mantle Cell Lymphoma in Adults
**Ciltacabtagene autoleucel**	**Cilta-cel**	• Adult Patients with Relapsed or Refractory Multiple Myeloma
**Idecabtagene vicleucel**	**Ide-cel**	• Adult Patients with Relapsed or Refractory Multiple Myeloma

Compared with traditional drugs, CAR-T therapy has shown remarkable efficacy in multiple clinical trials for the treatment of hematological malignancies (Maude et al., 2018; Abramson et al., 2020; Jacobson et al., 2022; Wang et al., 2023). Certain experimental data have demonstrated response rates and 1-year survival rates exceeding 60% and 50%, respectively (Schuster et al., 2019). Multiple drug regulatory agencies, including the FDA, have approved over 300 clinical trials of CAR-T cell therapy for both hematological and solid tumors. The use of CAR-T cells is expected to increase rapidly in the coming years, thereby benefiting more patients.

Although CAR-T therapy has shown promising results in numerous clinical trials, potential life-threatening or fatal toxicities cannot be ignored, such as cytokine release syndrome (CRS) and neurotoxicity (Neelapu et al., 2018). Rare adverse events (AEs), such as pulmonary or cardiovascular-related AEs, have also been reported (Alvi et al., 2019; Ganatra et al., 2020; Lefebvre et al., 2020). In a study evaluating the safety of CAR-T cells, 103 patients (8.53%) developed respiratory, thoracic, and mediastinal AEs. The most common type of AE in the respiratory system is hypoxia, which can lead to respiratory failure. Common comorbidities include dyspnea, pleural effusion, pulmonary edema, acute respiratory distress syndrome, and pneumonia (Chen et al., 2022). With multiple new CAR-T treatments approved in recent years, treatment choices may depend on product safety, in addition to efficacy.

Comprehensive studies on the potential toxicities associated with CAR-T therapy are currently lacking. In addition, clinical trials have inherent limitations in the detection of rare drug reactions. Consequently, spontaneous reports of adverse events have emerged as a vital source of information for identifying new safety signals, especially in post-market safety databases, such as FAERS. To effectively sift through these safety signals in a vast volume of reports, researchers have devised data-mining algorithms based on the concept of ‘disproportionality.’ This analysis detects statistical anomalies by comparing the reporting frequencies of a specific drug with those of all other drugs in a database (Michel et al., 2017). Common metrics used in disproportionality analysis include the Proportional Reporting Ratio (PRR), Reporting Odds Ratio (ROR), Information Component (IC), and Empirical Bayes Geometric Mean (EBGM). Recent publications have advocated the use of this approach (Wang et al., 2021; Yu et al., 2021; Tian et al., 2022; Yang et al., 2022; Chen et al., 2023), arguing that it can offer valuable insights to healthcare decision makers in formulating treatment strategies.

## 2 Materials and methods

### 2.1 Data sources

This was an observational, retrospective, pharmacovigilance study that utilized the FAERS database, a global repository of post-market safety reports. With SAS 9.4 and MedDRA 26.1, given the approval of the first CAR-T product in 2017, we decided to initiate our data collection from that year. Additionally, we chose to include data until the third quarter of 2023 as this represents the most recent and comprehensive dataset available to us. We analyzed the data from 2017 to 2023. The study screened reports within the database that contained any of the following trade or generic names of the drugs: “axicabtagene,” “ciloleucel,” “yescarta,” “axi-cel,” “KTE-C19,” “tisagenlecleucel,” “kymriah,” “tisa-cel,” “CTL019,” “brexucabtagene,” “autoleucel,” “tecartus,” “KTE-X19,” “lisocabtagene maraleucel,” “breyanzi,” “JCAR017,” “idecabtagene,” “vicleucel,” “abecma,” “bb2121,” “ciltacabtagene,” “autoleucel,” “carvykti” and “JCARH125,” This study utilized the FAERS database from Q1 2017 to Q2 2023.

### 2.2 Data deduplication

Spontaneous data collection often results in duplications or withdrawals/deletions from the database. The FDA’s official guidelines outline the rules for data deduplication and report deletion. This study strictly adhered to the data cleansing criteria. Initially, duplicate reports were removed using the FDA’s method: selecting PRIMARYID, CASEID, and FDA_DT from the DEMO table, and sorted by CASEID, FDA_DT, and PRIMARYID. Among identical CASEIDs, the report with the most recent FDA_DT is retained; if the CASEID and FDA_DT match, a higher PRIMARYID is chosen. Since Q1, 2019, the quarterly data packages have included deletion lists. After deduplication, the reports of CASEIDs on these lists were removed.

### 2.3 Data extraction

Adverse events (AEs) in the FAERS were coded using preferred terms (PTs) classified by MedDRA, which consists of 27 system organ classes (SOCs). All signal PTs and their corresponding SOCs were included, Specifically, “SOC = Respiratory, Thoracic, and Mediastinal Disorders” is designated as pulmonary adverse events (PAEs) in the context of this study. The clinical characteristics of CAR-T cell-related reports were extracted, including sex, age, reporting region, reporter, and indications. We also calculated the incidence of AEs caused by different CAR-Ts and the proportion of serious outcomes, defined as life-threatening or leading to hospitalization, disability, or death.

### 2.4 Data deduplication

During the data deduplication process, we first addressed duplicate entries by identifying and selecting the most recent and comprehensive records based on the CASE and FDA_DT fields (i.e., records with the latest FDA_DT or a higher ISR number). Subsequently, we focused on data inconsistencies, validating the values of each field, and deciding whether to retain, correct, or delete abnormalities. Additionally, missing values were appropriately handled by either filling or deleting depending on their significance and impact on the overall dataset. Furthermore, the integrity of the data was ensured by verifying each step of the cleaning process and comparing it with the original data.

### 2.5 Data mining

Disproportionality analysis was conducted to detect potential signals of AEs caused by CAR-T therapy. This analysis compared the proportion of CAR-T cell therapy AEs reports with the proportion of reports on all other drugs. Algorithms such as the ROR, PRR, IC, and EBGM were used. The equations and corresponding thresholds for the algorithms are listed in Table 2. Only AEs with signals detected by all four algorithms were included in this study.

**TABLE 2:** Algorithms used for signal detection.

Algorithms	Equation	Criteria
ROR	$\text{ROR}=\frac{\left(a/c\right)}{\left(b/d\right)}=\frac{ad}{bc}$	lower limit of 95% CI > 1, N ≥ 3
	95%CI = eln (ROR)±1.96 (1/a+1/b+1/c+1/d)^0.5	
PRR	$\text{PRR}=\frac{a/\left(a+b\right)}{c/\left(c+d\right)}$	PRR ≥ 2 and χ2≥ 4, N ≥ 3
	$\chi 2=\frac{\;{\left(\text{ad}-\text{bc}\right)}^{2}\left(a+b+c+d\right)}{\left(\;a+b\right)\left(a+c\right)\left(c+d\right)\left(b+d\right)}$	
IC	IC = $\log_{2}\frac{a\left(a+b+c+d\right)}{\left(a+b\right)\left(a+c\right)}$	lower limit of 95% CI > 0, N ≥ 3
	95%CI = E (IC) ± 2V(IC)^0.5	
EBGM	$\text{EBGM}=\frac{a\left(a+b+c+d\right)}{\left(a+c\right)\left(a+b\right)}$	lower limit of 95% CI > 2, N ≥ 3
	$95\%\text{CI}=e^{\ln\left(\text{EBGM}\right)\pm 1.96\sqrt{\;\left(\frac{1}{a}+\frac{1}{b}+\frac{1}{c}+\frac{1}{d}\right)\;}}$	

Equation: a: number of reports containing both the target drug and target adverse drug reaction

b: number of reports containing other adverse drug reaction of the target drug

c: number of reports containing the target adverse drug reaction of other drugs

d: number of reports containing other drugs and other adverse drug reactions

95%CI, 95% confidence interval; ROR, Reporting Odds Ratio; PRR, Proportional Reporting Ratio; IC, Information Component; EBGM, Empirical Bayes Geometric Mean.

## 3 Results

### 3.1 Descriptive analysis

**FIGURE 1:**
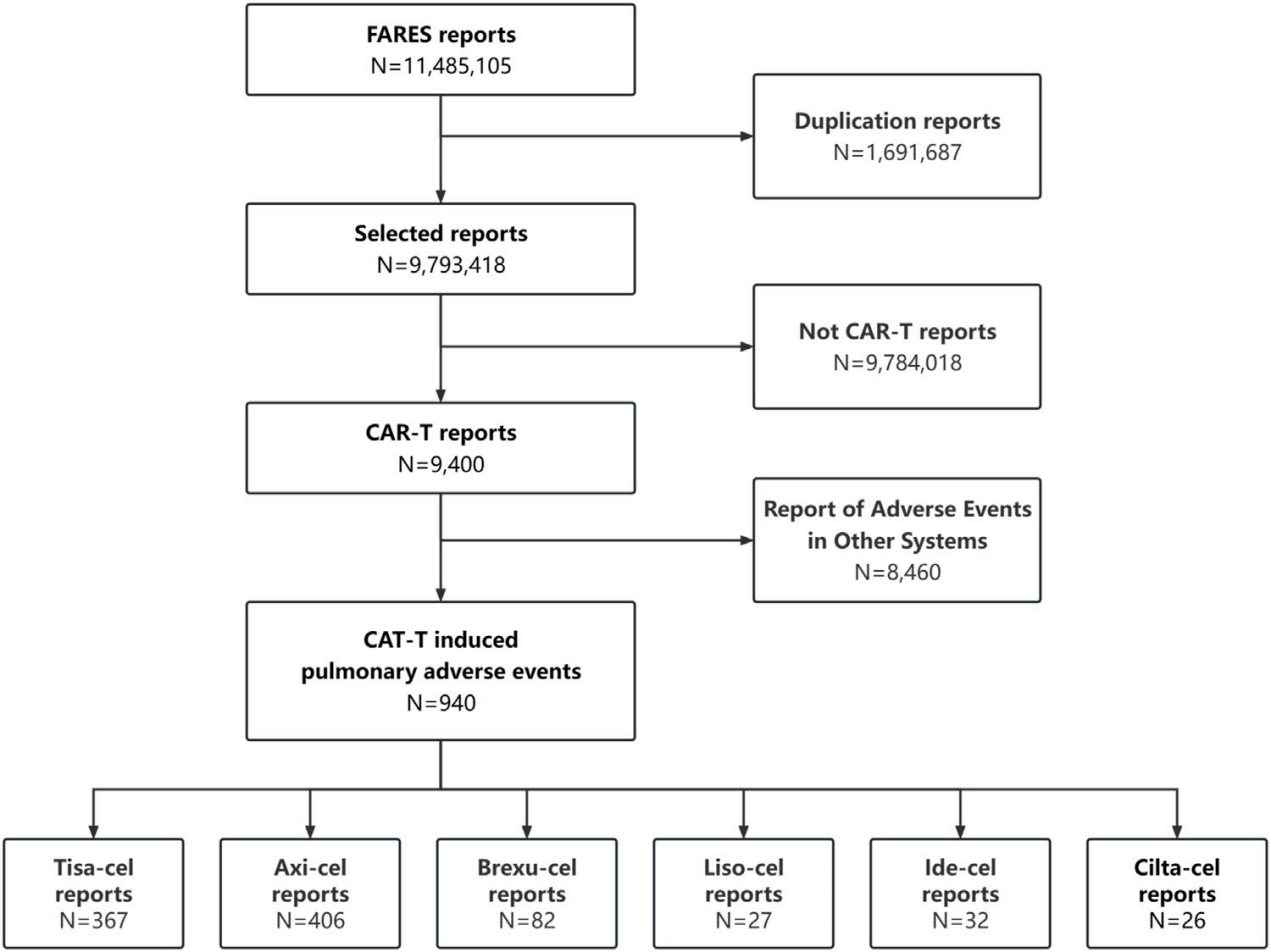
The main steps in the processing of the FAERS database.

Between January 2017 and September 2023, 9,400 CAR-T-related AE were recorded, including 940 PAEs (10%), with some patients potentially experiencing multiple occurrences. The retrieval process is illustrated in Figure 1.

**FIGURE 2:**
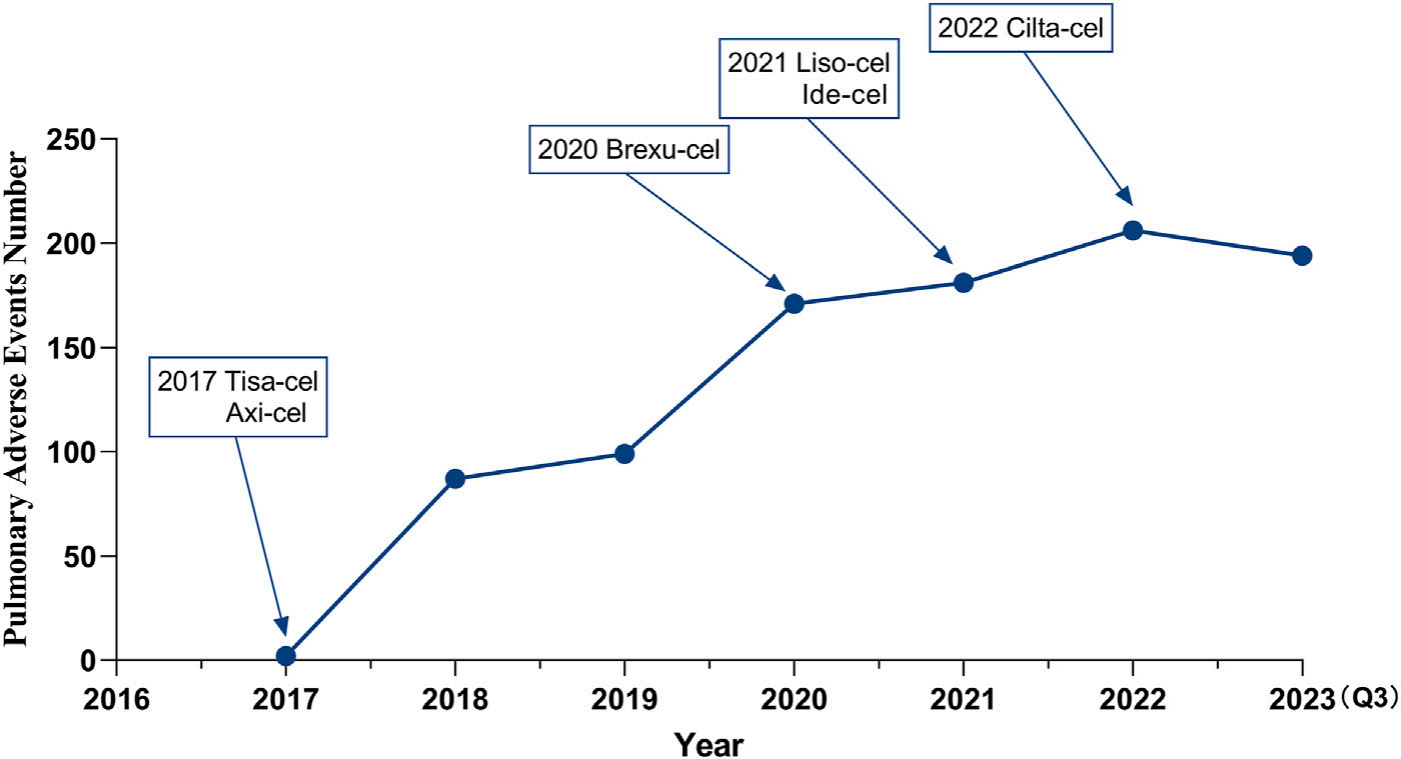
The number of CAR-T-related PAEs reported to the FAERS each year.

With the sequential approval of CAR-T therapy, the incidence of PAEs has increased annually (Figure 2), and males were found to experience these events more frequently (538 cases, 57.23%) than females (350 cases, 37.23%). Most reported patients were between 18 and 65 years of age, with a median age of 59 years. These reports originated primarily in the United States (64.04%), followed by France (3.40%). The number of reports on this topic is increasing annually. More than 80% of the reports were submitted by healthcare professionals, including 41.60% physicians (Table 3).

**TABLE 3:** Main characteristics of the CAR-T reports.

Clinical characteristics	Patients with PAEs (940	Total (9400)
Gender		
Female (%)	350 (37.23)	2853 (30.35)
Male (%)	538 (57.23)	4671 (49.69)
Missing (%)	52 (5.53)	1876 (19.96)
Age		
<18 (%)	132 (14.04)	577 (6.14)
≥18, <65 (%)	383 (40.75)	3278 (34.87)
65≤ (%)	302 (32.13)	2496 (26.56)
Missing (%)	123 (13.09)	3049 (32.44)
Media (IQR)	59 (30,69)	61 (46,69)
Country		
United States(%)	602 (64.04)	6021 (64.05)
France (%)	32 (3.40)	391 (4.16)
Spain (%)	16 (1.70)	295 (3.14)
Germany (%)	26 (2.77)	291 (3.10)
Italy (%)	7 (0.74)	230 (2.45)
Other (%)	257 (27.34)	2172 (23.11)
Reporter type		
Healthcare profession (%)	754 (84.81)	7756 (82.51)
Physician (%)	391 (41.60)	3679 (39.14)
Pharmacist (%)	307 (32.66)	3261 (34.69)
Other health-professional (%)	96 (10.21)	816 (8.68)
Consumer (%)	72 (7.66)	965 (10.27)
Missing (%)	74 (7.87)	679 (7.22)

### 3.2 Disproportionality analysis

#### 3.2.1 Signal values associated with CAR-T therapies

Figure 2 shows the signal intensity of the CAR-T cell-related PAEs. Hypoxia showed the highest number of reports (n = 344) and the strongest signal in the AE signal calculation method (IC = 4.09, ROR = 17.54), as well as a large number of reports of respiratory failure (n = 127) with strong signals (IC = 1.69, ROR = 3.25).

**FIGURE 3:**
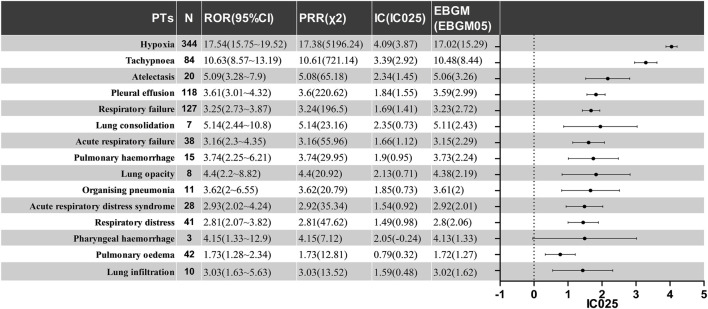
The positive signal distribution of CAR-T treatment for PAEs using standardized MedDRA queries. PT, preferred term; ROR, reporting odds ratio; CI, confidence interval; PRR, proportional reporting ratio; χ2, chi-information component; IC, information component; IC025, the lower limit of 95% CI of the IC; EBGM, empirical bayesian geometric mean; EBGM05, the lower limit of 95% CI of EBGM.

Other PAEs include Pleural effusion (IC = 1.84, ROR = 3.61), Acute respiratory failure (IC = 1.66, ROR = 3.16), Pulmonary hemorrhage (IC = 1.9, ROR = 3.74), Acute respiratory distress syndrome (ARDS) (IC = 1.54, ROR = 2.93), Organizing pneumonia (IC = 1.85, ROR = 3.62), Respiratory distress (IC = 1.49, ROR = 2.81), Pharyngeal hemorrhage (IC = 2.05, ROR = 4.15), and others. Furthermore, all detected safety signals must be confirmed using all four methods, including the PRR and EMBG. The complete data are shown in Figure 3.

#### 3.2.2 Analysis of the quantity and distribution of PAEs in CAR-T therapies

**FIGURE 4:**
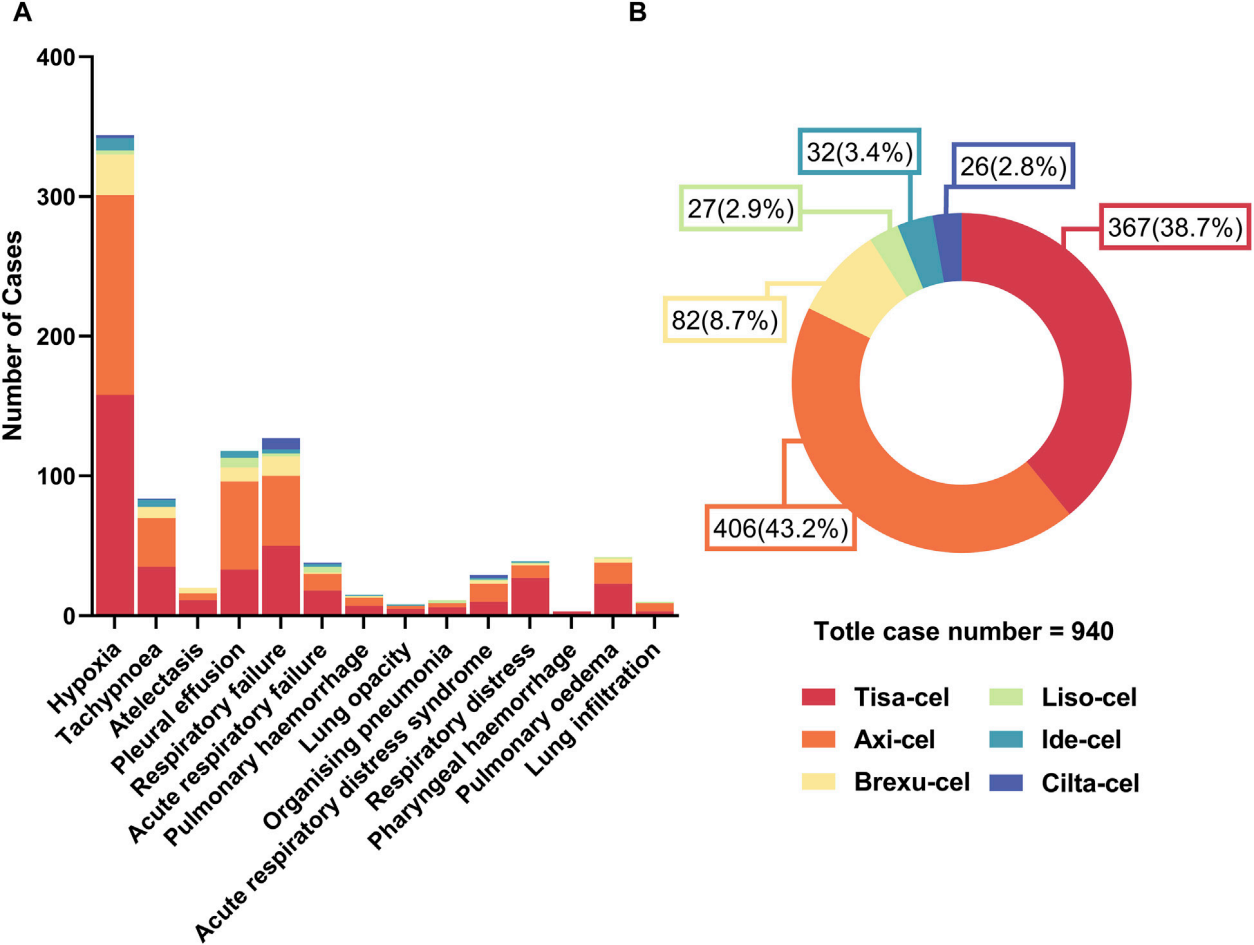
Quantitative overview of adverse events in car-t therapies. **(A)** Number of different types of PAEs across car-t therapies. **(B)** Proportional distribution of PAEs among car-t therapies.

As shown in Figure 4A, Hypoxia and Respiratory failure were the most frequently occurring PAEs in all CAR-T therapies, significantly outnumbering the other types. Notably, Pleural effusion is also a prominent issue, especially with Tisa-Cel and Axi-Cel. Although Atelectasis, Acute respiratory failure, and acute respiratory distress syndrome are less common, they still occur. Analysis of the data in Figure 4B revealed 940 adverse respiratory events following six CAR-T cell treatments. Tisa-Cel and Axi-Cel were associated with higher adverse event totals. Specifically, Tisa-Cel had 367 events, including 158 hypoxic and 50 respiratory failure cases. Axi-Cel reported 406 events, with 143 hypoxia and 50 respiratory failure instances. Conversely, Cilta-Cel demonstrated superior respiratory safety with only 26 reported events. Brexu-Cel, Ide-Cel, and Liso-Cel resulted in 82, 32, and 27 events, respectively. Respectively.

#### 3.2.3 Analysis of signal spectra of PAEs following different CAR-T therapies

**FIGURE 5:**
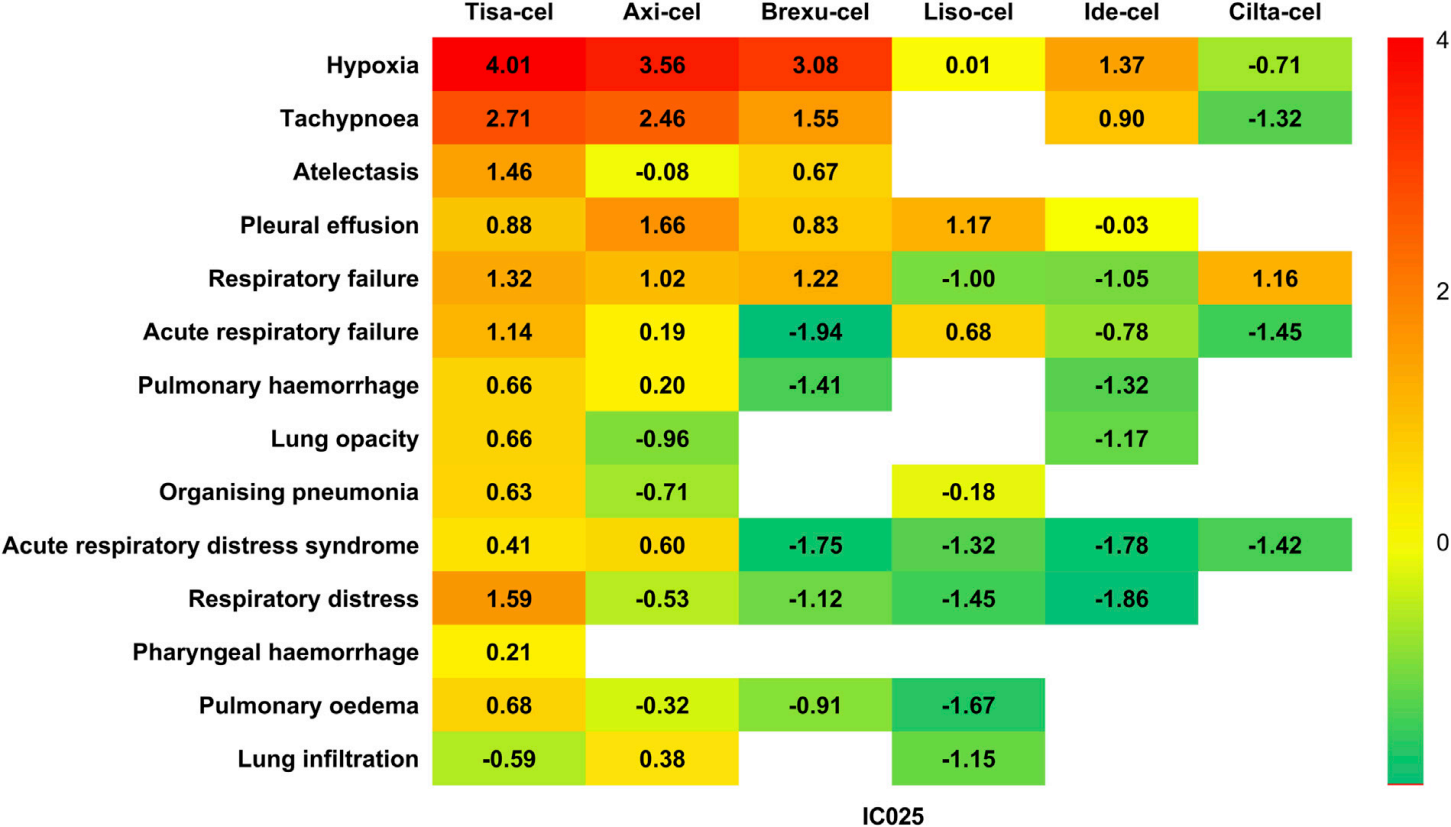
Heatmap for safety signals based on the lower limit of the 95% confidence interval of the information component (IC025).

We further analyzed the signals of specific AEs across the different CAR-T cell therapies. Reports with fewer than 3 cases were excluded to prevent false positives. Using the lower limit of the 95% confidence interval of the information component (IC025) as a metric, we created a heat map for safety signals based on IC025 (Figure 5). Based on the aforementioned results, we determined that an IC025 greater than 0 constituted a safety signal, indicating a stronger correlation between the drug or treatment and a specific AE with increasing values.

When considering different therapies individually, Tisa-cel has been found to be associated with the highest diversity of AEs. Among the 19 PTs detected, 13 demonstrated safety signals, including serious clinical AEs, such as respiratory failure (IC025 = 1.32), acute respiratory failure (IC025 = 1.14), pulmonary hemorrhage (IC025 = 0.63), lung opacity (IC025 = 0.66), and ARDS (IC025 = 0.41). Additionally, Tisa-Cel demonstrated strong safety signals in hypoxia (IC025 = 4.01) and tachypnea (IC025 = 2.71).

Axi-cel has been found to be associated with nine AEs, with relatively strong associations in hypoxia (IC025 = 3.56) and tachypnea (IC025 = 2.46), and also demonstrating safety signals in pleural effusion (IC025 = 1.66) and respiratory failure (IC025 = 1.02).

Brexu-cel has been found to be associated with five AEs, including respiratory failure (IC025 = 1.22) and pleural effusion (IC025 = 0.83).

Liso-cel showed safety signals for the three AEs, with the strongest association with pleural effusion (IC025 = 1.71).

Ide-Cel was only been found to be associated with hypoxia (IC025 = 1.37) and tachypnea (IC025 = 0.90).

Interestingly, the safety signal spectrum of Cilta-Cel was distinct from those of other CAR-T therapies. It does not exhibit a correlation with the commonly observed AEs of hypoxia and tachypnea, and instead shows a safety signal solely in respiratory failure (IC025 = 1.16) among all AEs.

### 3.3 Time to onset

**FIGURE 6:**
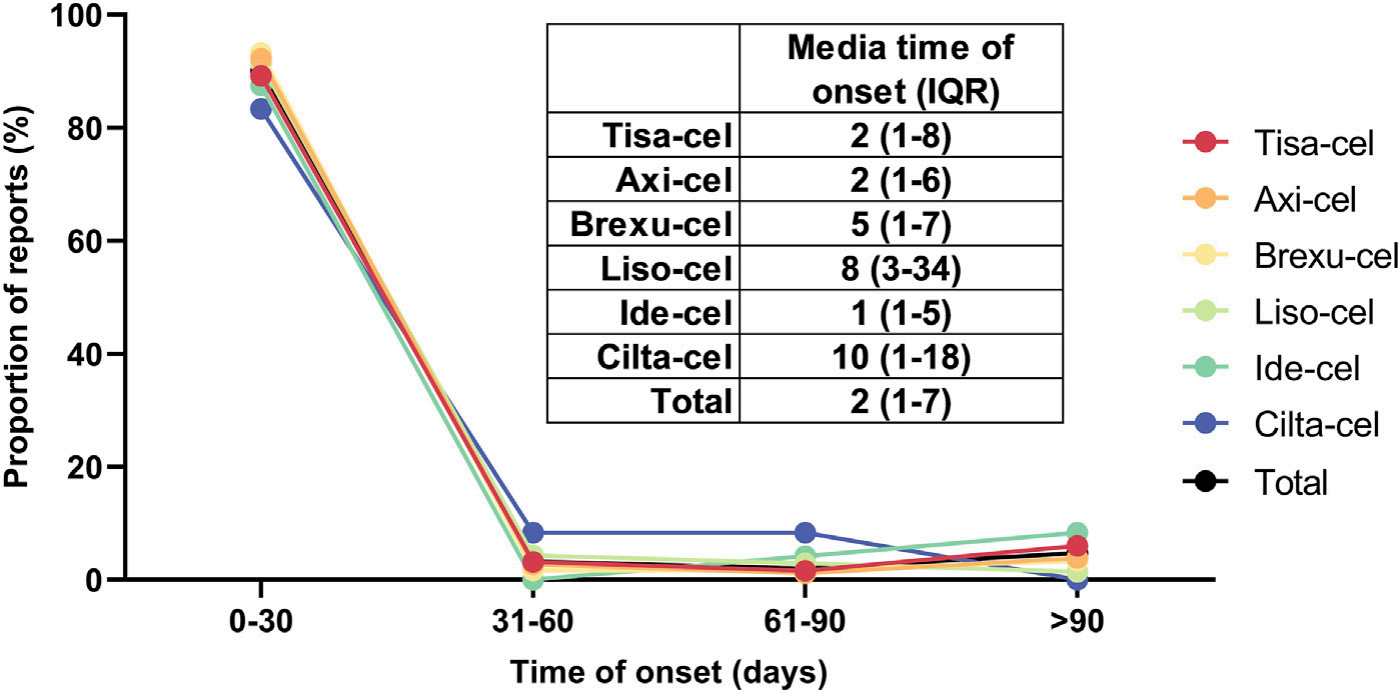
Days from CAR-T infusion to adverse event onset.

Figure 6 illustrates the time difference between the initiation of CAR-T treatment and the occurrence of PAEs, including the overall median and interquartile range (IQR) of the time of onset of these events after each type of CAR-T treatment. No significant differences were observed in the incidence of AEs between the CAR-T therapies. Most adverse reactions occurred within 0–30 days, with Cilta-Cel exhibiting the longest average onset time of 10 days (IQR: 1–18). The median onset time for AEs of other CAR-T therapies was between 1–8 days. After integrating and analyzing the data, we found that the majority of PAEs following CAR-T cell therapy occurred within 30 days of treatment, comprising 89.97% of all reported cases. Furthermore, the median time to onset of these events was 2 days, with an interquartile range (IQR) of 1–7 days.

### 3.4 Fatality proportion

**FIGURE 7:**
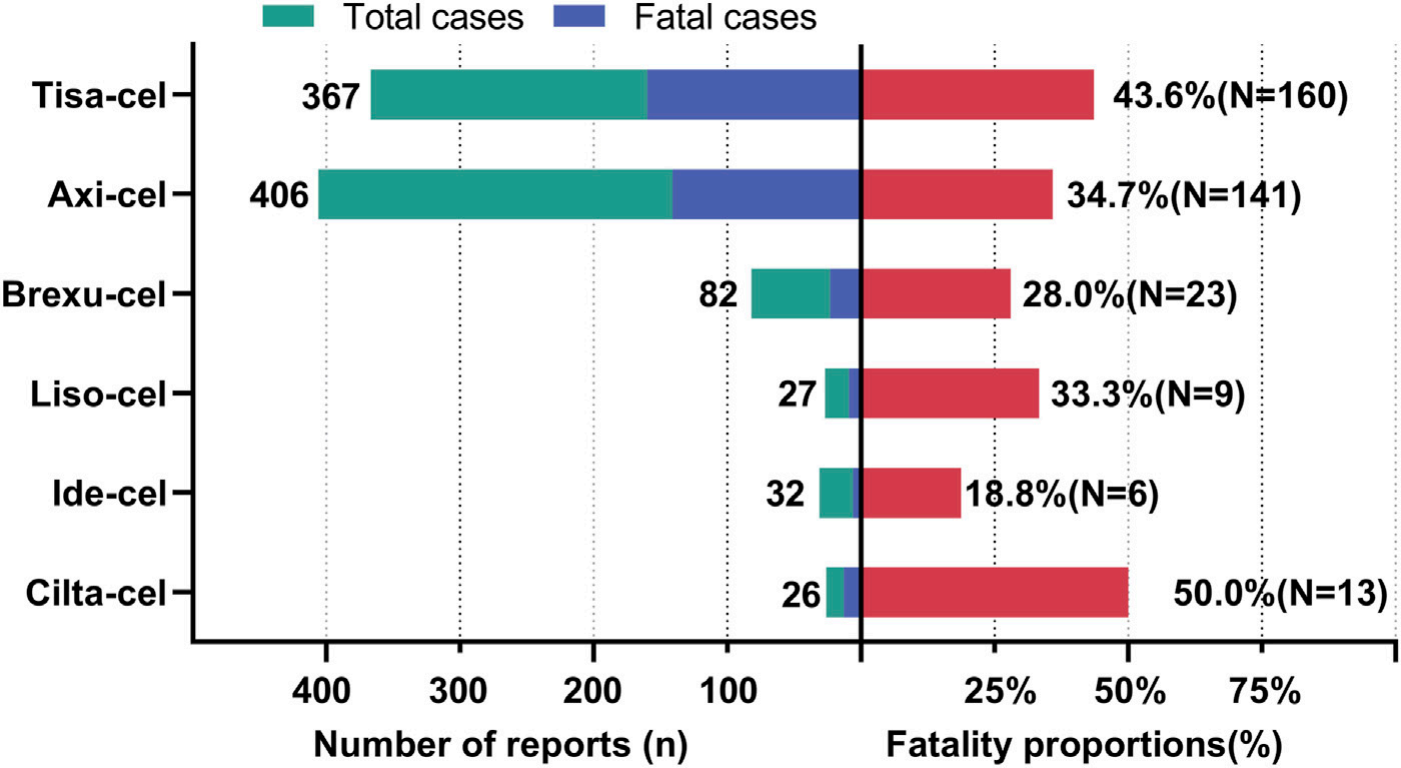
Fatalities due to PAEs from various CAR-T therapies.

Figure 7 shows the fatality rates of PAEs following CAR-T cell therapy. According to database statistics, Tisa-cel had the highest number of deaths (160 out of 367 cases, 43.6%) and Axi-cel had the highest incidence of PAEs (406 cases) among all CAR-T therapies, with 141 patients (34.7%) experiencing death. Among the 32 cases of PAEs associated with Ide-cel, only six resulted in death, representing the lowest mortality rate of 18.8%. The mortality rates of the PAEs associated with Brexu-cel and Liso-cel were 28.0% and 33.3%, respectively. Finally, Cilta-cel had 26 cases of PAEs related to it, of which 13 resulted in death, with a mortality rate of 50%. Because of the short time since its market release and the limited overall sample size, the relatively high fatality rate observed may be influenced by “small sample bias.” It is necessary to conduct further analyses with a larger sample size in the future to more accurately assess the fatality rate.

## 4 Discussion

### 4.1 Overview of PAEs associated with CAR-T therapy

In recent years, CAR-T cell therapy has demonstrated remarkable therapeutic efficacy in the field of antitumor treatment; however, safety concerns have become increasingly prominent. To the best of our knowledge, this is the first comprehensive analysis of PAEs associated with CAR-T therapy.

From January 2017 to September 2023, 9,400 CAR-T related AE reports were documented, with PAEs accounting for 940 cases (10%), indicating a significant incidence of PAEs associated with CAR-T therapy. With the widespread use of this therapy, the annual incidence of PAEs has increased, posing a major safety concern. Male patients (57.23%) were more prone to these events than female patients (37.23%), suggesting that factors such as physiological and immune system characteristics, drug metabolism, lifestyle habits, and smoking history affect the occurrence of PAEs in CAR-T therapy. The median age of the patients was 59 years, mainly in the age range of 18–65 years old, representing middle-aged individuals. This age group exhibits a gradual decline in physiological functions, including the decreased functionality of the lungs, heart, liver, and kidneys. In addition, they experience hormonal changes that are often accompanied by the emergence of chronic diseases. This physiological decline may affect drug metabolism and the efficacy of CAR-T therapy, leading to variations in the incidence of PAEs among individuals (Kaddu-Mulindwa et al., 2022). Regarding the source of reports, the majority of the reports originate from the United States (64.04%), followed by France (3.40%). Differences in reporting sources are believed to be primarily attributable to disparities in the development of healthcare systems and regulatory frameworks among countries; thus, they cannot be directly linked to geographical differences. More than 80% of the reports were submitted by healthcare professionals, emphasizing their crucial role in monitoring and reporting the safety of CAR-T cell therapy and ensuring the credibility of the reports.

### 4.2 CAR-T therapy and the causal link to PAEs

In CAR-T cell therapy, CRS is one of the most common adverse reactions, with symptoms ranging from mild infusion reactions and fever to systemic symptoms, such as hypotension (Murthy et al., 2019; Sterner et al., 2019), hypoxia, capillary leak, and organ dysfunction (Srivastava et al., 2019; Castellarin et al., 2020; Labanieh et al., 2022). The consensus criteria set by the American Society for Transplantation and Cellular Therapy (ASTCT) indicate a direct correlation between the severity of CRS and degree of hypoxic symptoms (Lee et al., 2019). The MSKCC team identified objective factors for distinguishing CRS severity in early clinical trials and established a corresponding grading system. Notably, hypoxia is a pivotal factor, with an FiO2 of 40% marking the boundary between grades 2 and 3, and the need for intubation is defined as grade 4 CRS (Park et al., 2018). Our study showed that hypoxia was the most common pulmonary AE following CAR-T therapy, which is consistent with previous research and clinical practice (Shalabi et al., 2020).

During CAR T-cell therapy, genetically modified cells specifically target and eliminate tumor cells. In this process, cytokines such as IFN-γ and TNF-α are released, activating the body’s endogenous immune cells and initiating a series of inflammatory reactions. The lungs, with their unique physiological structure characterized by a thin endothelial cell layer and a dense vascular network, are prime sites for the accumulation and action of these inflammatory mediators. As cytokines accumulate and CAR-T cells proliferate, inflammatory factors infiltrate the lung microvasculature and cause endothelial cell damage. Ultimately, this results in compromised lung function, tissue edema, and a spectrum of subsequent adverse reactions ranging in severity.

### 4.3 Manifestations of CAR-T therapy-associated PAEs

Patients with lung injury commonly experience hypoxia and tachypnea; however, specific AEs are associated with certain CAR-T treatments. Tisa-cel, Axi-cel, and Brexu-cel showed stronger associations with respiratory failure and distress than the other therapies. Although some researchers attribute severe PAEs primarily to CRS, alternative views exist (Lichtenstein et al., 2021; Chohan et al., 2023). In a study of CAR-T cells targeting high-activity mesothelin (MSLN), two patients developed progressive hypoxemia within 48 h of infusion, aligning with CRS clinically and laboratorily. One patient died of respiratory failure, and autopsy revealed acute lung injury, widespread T-cell infiltration, and CAR T-cell accumulation in the lungs. Upregulated MSLN expression in damaged lung cells may lead to dose-limiting toxicity (Haas et al., 2023), suggesting that CAR-T cell therapy may induce targeted tissue damage owing to dynamic antigen expression in normal cells. Vigilant monitoring is crucial for mitigating risks during treatment.

Our study revealed a correlation between CAR-T therapy and AEs diagnosed through imaging examinations, including pleural effusion, atelectasis, lung consolidation, and organizing pneumonia. Compared with AEs based on subjective experiences, such as hypoxia and tachypnea, these events exhibit lower numbers and signal intensities. This discrepancy can be attributed to the diagnostic challenges associated with imaging, which may lead to the underestimation or oversight of mild or ambiguous cases. Physicians also tend to prioritize reporting AEs based on subjective experiences given their accessibility to direct patient perceptions and descriptions. Current studies indicate that specific findings from imaging modalities, such as CT, play a pivotal role in the early detection and prompt intervention of AEs, emphasizing the crucial significance of PET/CT in managing AEs following CAR-T therapy (Smith et al., 2022), The establishment of standardized imaging examination mechanisms is imperative in clinical practice.

### 4.4 Onset timing and fatality rate of PAEs

We observed variations in the reported mortality rates across the different CAR-T therapies. Tisa-Cel, which has been associated with 159 fatalities, had the highest death toll, raising concerns. The increased number of deaths could potentially be attributed to factors such as target specificity, cell expansion dynamics, duration of persistence, and cytokine storms. Moreover, it is essential to recognize that the extended market duration and extensive patient population of Tisa-Cel could potentially lead to an increased number of reported fatalities. Although axi-cell therapy is associated with the highest number of PAEs, its relatively low mortality rate may suggest some advantages in managing PAEs. However, this does not imply that Axi-Cel is entirely safe for lung function, as a substantial number of AE reports still emphasize the need for further optimization. Despite the limited number of cases, the high mortality rate associated with Cilta-Cel therapy is noteworthy. Given the Cilta-Cel ‘s relatively recent market entry, this could be due to its initial inexperience or other unknown factors, necessitating deeper evaluations and investigations into its safety profile. In addition, we believe that the voluntary nature of reporting in the FAERS database results in certain AEs, particularly moderate and severe AEs, that receive heightened attention and extensive reporting owing to their salience and severity. Conversely, mild events may be overlooked or inadequately reported because of a lack of salience or insufficient attention. This phenomenon of selective reporting contributes to the emergence of information bias, which is a significant factor accounting for the unusually high mortality rates and other adverse outcomes associated with such adverse events in the database.

We obtained some findings regarding the time of occurrence of AEs. Nearly 80% of CAR-T cell-related PAEs occur within 30 days, mainly due to immune responses and cytokine cascades resulting from interactions between CAR-T cells and tumor cells, thereby increasing the risk of Cytokine Release Syndrome (CRS), we firmly advocate that prompt identification and immediate intervention are essential for managing short-term Adverse Events (AEs), which must be accomplished through rigorous monitoring at the initiation of treatment. However, monitoring discrepancies might affect the initially high incidence, and a decrease in monitoring frequency over time could lead to missed diagnoses of mild or asymptomatic events. We investigated the differences in pulmonary AE onset times among the six CAR-T cell treatments but found no significant variations. This minor similarity suggests a shared level of lung safety, which is potentially linked to common treatment mechanisms, components, and strategies. However, limitations in data collection and analysis, such as an insufficient sample size for detecting subtle distinctions or measurement biases, lead to high consistency and caution against definitive conclusions. Further research is required to validate and enhance our understanding of these observations.

## 5 Limitations

Data from the FAERS database may be incomplete or missing, thus impeding the comprehensive assessment of the safety of CAR-T therapy. Patient data were mainly obtained from the USA, potentially leading to ethnic and regional disparities. Moreover, most patients in this study had received prior anticancer treatments, which might have influenced the safety evaluations of CAR-T therapy. Disproportionality analysis also has significant limitations in drug safety assessments. This method relies heavily on spontaneously reported data, which lack accurate information on reaction incidence rates, thus impeding the comprehensive assessment of adverse drug reactions. Furthermore, the presence of reporting biases and confounding factors may severely affect the analytical results, thereby diminishing their accuracy. Therefore, a disproportionality analysis can only provide preliminary hypotheses and must be combined with other methodologies to ensure the accuracy and comprehensiveness of the evaluation.

## 6 Conclusion

This study comprehensively described the PAEs reported in the FAERS database for patients receiving CAR-T cell therapy, including conditions such as hypoxia, respiratory failure, pleural effusion, and atelectasis. These events are associated with a higher incidence and risk in patients undergoing CAR-T cell therapy, can severely impact breathing, and are life-threatening. To minimize these risks, physicians should consider preconditioning drugs, fine-tune CAR-T doses, and alternative treatment plans. Lung issues associated with CAR-T therapy are clinically significant and require attention from both doctors and researchers.

## Data Availability

The original contributions presented in the study are included in the article/Supplementary Material, further inquiries can be directed to the corresponding authors.
